# Increased Serum Levels of Growth-Differentiation Factor 3 (GDF3) and Inflammasome-Related Markers in Pregnant Women during Acute Zika Virus Infection

**DOI:** 10.3390/v14051004

**Published:** 2022-05-09

**Authors:** Carlos Eduardo de Castro Alves, Sabrina Araújo de Melo, Jean de Melo Silva, Leonardo Calheiros de Oliveira, Valdinete Alves do Nascimento, João Hugo Abdalla Santos, Felipe Gomes Naveca, Gemilson Soares Pontes

**Affiliations:** 1 Programa de Pós-Graduação em Imunologia Básica e Aplicada—PPGIBA, Instituto de Ciências Biológicas, Universidade Federal do Amazonas—UFAM, Manaus 69080-900, Brazil; alveseduardo71@gmail.com (C.E.d.C.A.); biomedicojean@gmail.com (J.d.M.S.); 2 Laboratório de Virologia e Imunologia, Instituto Nacional de Pesquisas da Amazônia, Manaus 69060-001, Brazil; sabrinaamello65@gmail.com (S.A.d.M.); leocalheiros7@gmail.com (L.C.d.O.); 3 Laboratório de Ecologia de Doenças Transmissíveis na Amazônia, Instituto Leônidas e Maria Deane, Fiocruz, Manaus 69057-070, Brazil; val.alvesnascimento@gmail.com (V.A.d.N.); felipe.naveca@fiocruz.br (F.G.N.); 4 Hospital Adventista de Manaus, Manaus 69075-351, Brazil; jh_abdalla@yahoo.com.br; 5 Laboratório de Flavivírus, Instituto Oswaldo Cruz, Fiocruz, Rio de Janeiro 21040-360, Brazil

**Keywords:** ZIKV, pregnancy, NLRP3, IL-1β, IL-18, GDF3, inflammasome

## Abstract

The systemic inflammatory response elicited by acute Zika virus (ZIKV) infection during pregnancy plays a key role in the clinical outcomes in mothers and congenitally infected offspring. The present study aimed to evaluate the serum levels of GDF-3 and inflammasome-related markers in pregnant women during acute ZIKV infection. Serum samples from pregnant (*n* = 18) and non-pregnant (*n* = 22) women with acute ZIKV infection were assessed for NLRP3, IL-1β, IL-18, and GDF3 markers through an enzyme-linked immunosorbent assay. ZIKV-negative pregnant *(n* = 18) and non-pregnant women (*n* = 15) were used as control groups. All serum markers were highly elevated in the ZIKV-infected groups in comparison with control groups (*p* < 0.0001). Among the ZIKV-infected groups, the serum markers were significantly augmented in the pregnant women in comparison with non-pregnant women (NLRP3 *p* < 0.001; IL-1β, IL-18, and GDF3 *p* < 0.0001). The IL-18 marker was found at significantly higher levels (*p* < 0.05) in the third trimester of pregnancy. Bivariate and multivariate analyses showed a strong positive correlation between GDF3 and NLRP3 markers among ZIKV-infected pregnant women (r = 0.91, *p* < 0.0001). The findings indicated that acute ZIKV infection during pregnancy induces the overexpression of GDF-3 and inflammasome-related markers, which may contribute to congenital disorders and harmful pregnancy outcomes.

## 1. Introduction

Zika virus (ZIKV) is an arthropod-borne flavivirus that may cause illness of high morbidity and mortality in humans, which poses unique challenges for the public health and economy of many countries [1]. In general, ZIKV infection causes a broad variety of non-specific clinical manifestations [2]. However, during pregnancy, ZIKV infection is associated with congenital brain malformations, placental insufficiency, intrauterine growth restriction, and even fetal death [3,4].

The tropism of ZIKV for fetal embryonic and human neural progenitor cells associated with the host immune response are responsible for the clinical spectrum of disease presentation in the context of pregnancy [5,6]. Brain endothelial and neuroglia cells are persistent reservoirs of ZIKV, which may contribute to central nervous system (CNS) inflammation. This inflammatory condition is characterized by elevated serum levels of immune mediators such as fibroblast growth factor basic (FGF-basic), *platelet-derived growth factor* (PDGF), vascular endothelial growth factor (VEGF), interleukin-1 beta (IL-1β), interleukin-18 (IL-18), and interferon gamma-induced protein 10 (IP-10) [6,7,8].

Even though the upregulation of inflammatory mediators drives the development of severe pregnancy-related complications, the clinical meaning of growth factors-ZIKV interaction is unknown [9]. Growth factors mediate a myriad of cellular processes by regulating cell survival through the balance between proliferation, growth, and death [10]. For example, the growth differentiation factor-3 (GDF3), a protein of the factor-β (TGF-β) superfamily, regulates the early embryonic development and the process of lipolysis [11,12]. However, the expression profile of growth factors during pregnancy in the context of ZIKV infection and its implication on pregnancy outcomes are still unclear.

ZIKV infection also induces the expression of inflammasome marker NOD-like receptor protein 3 (NLRP3), which prevents the interferon type 1 (IFN-1) antiviral response [13]. The inflammasome is a multiprotein complex composed of innate important components that coordinate the inflammatory response and play a critical role in the homeostasis of different tissues [14]. Balancing inflammation is essential throughout all steps of pregnancy, especially during the early and late gestational period [15,16]. For example, the upregulation of inflammasome markers is associated with poor pregnancy outcomes, such as preeclampsia and pre-term labor [17].

The early expression of NLRP3 and its activation in the placenta can lead to many pregnancy complications [18]. Although gestational tissues usually express inflammasomes, the magnitude of NRLP3 and its downstream mediators’ expression induced by ZIKV infection in the context of pregnancy is not known. Thus, this study evaluated the serum levels profile of GDF3 and inflammasome-related markers in pregnant women during acute ZIKV infection.

## 2. Materials and Methods

### 2.1. Ethics Statement

The study was approved by the Ethics Committee of the Universidade do Estado do Amazonas (CAAE: 56745116.6.0000.5016). All individuals signed the informed consent form before taking part in this study. Informed consent was obtained from a parent and/or legal guardian of the participants under the age of 18 years old. Confidentiality and the right to leave the study at any time were guaranteed to all participants. The experiments were performed under relevant guidelines and regulations.

### 2.2. Study Population

From March to December 2016, 73 women were recruited through a non-probabilistic convenience sampling strategy at different outpatient public health care facilities in Manaus, Amazonas, Brazil. The study population was composed of 36 pregnant women (at the 1st to 3rd trimester of pregnancy; aged 15–37 years old) and 37 non-pregnant women (aged 27 to 50 years old) with diagnostic positive or negative for ZIKV infection. Molecular diagnostic of ZIKV infection (envelope coding region) was performed at the Leonidas and Maria Deane Institute (Fiocruz-AM) using an RT-qPCR test assay, as previously described [19]. After molecular confirmation of ZIKV infection, the study population was stratified as the following: ZIKV-infected pregnant (Pr ZIKV+; *n* = 18) and non-pregnant (NPr ZIKV+; *n* = 22) women; ZIKV-negative pregnant (Pr ZIKV−; *n* = 18) and non-pregnant women (NPr ZIKV−; *n =* 15).

### 2.3. Analysis of Serum Markers

We evaluated the serum concentration of the GDF3, NLRP3, IL-1β, and IL-18 using enzyme-linked immunosorbent assay according to the manufacturer’s instructions (Wuhan Fine Biotech CO., Wuhan, China; Thermo Fisher Scientific, Waltham, USA). Results for NLRP3 were expressed in ng/mL and for IL-1β, IL-18, and GDF3 in pg/mL. All serological tests were performed at the Laboratory of Virology and Immunology of the National Institute of Amazonian Research, Manaus, Brazil.

### 2.4. Statistical Analysis

One-Way ANOVA, followed by Tukey’s post-hoc test, was used to evaluate the difference of biomarkers’ concentrations observed between the study groups. Outliers were identified based on interquartile range calculation (IRQ) and excluded from the analysis to avoid the *masking* effects. Spearman’s test was applied to assess the correlation between the biomarkers in the ZIKV-infected groups. The correlation was considered strongly positive when r > 0.7. We performed all statistical analyses by using GraphPad Prism v8.0.1 software and Gephi v0.9.2 software. In all analyses, a value of *p* < 0.05 was considered statistically significant.

## 3. Results

### 3.1. ZIKV Infection Upregulates the GDF3, NLRP3, IL-1β and IL-18 Expression in Pregnant Women

**Figure 1:**
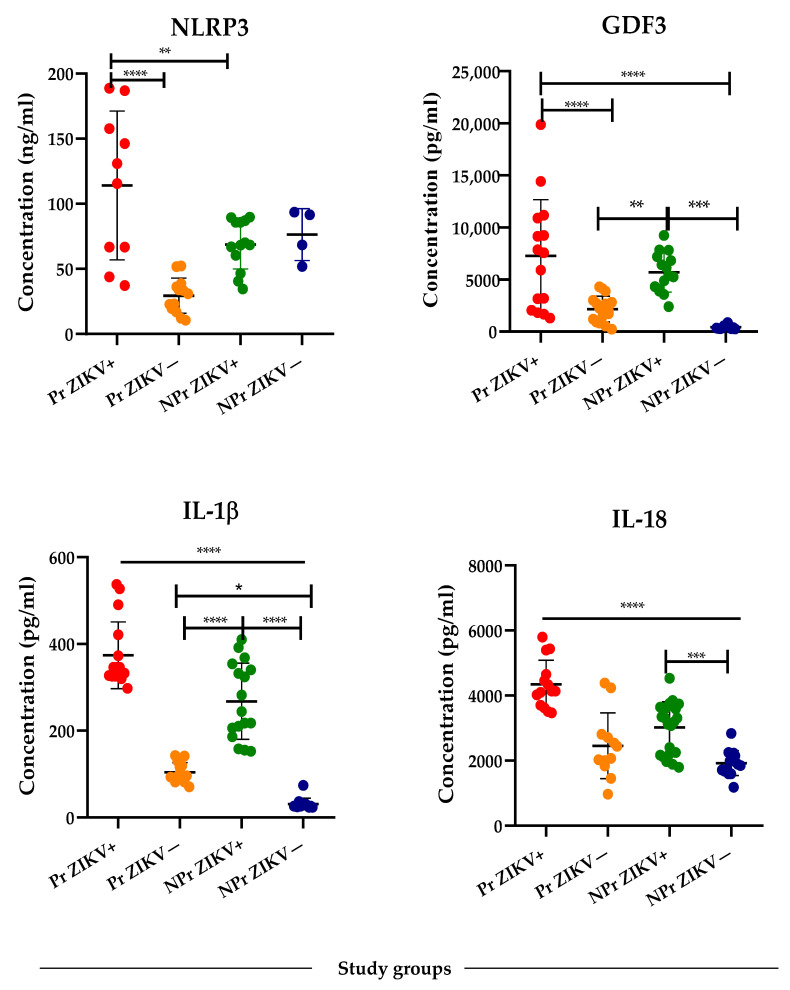
Serum levels of GDF3 and inflammasome-related markers. Analysis of serum marker levels performed in ZIKV-infected pregnant women (Pr ZIKV+), ZIKV-negative pregnant women (Pr ZIKV−), ZIKV-infected non-pregnant women (NPr ZIKV+), ZIKV negative non-pregnant women (NPr ZIKV−). One-Way ANOVA with Tukey’s multiple comparison test was performed in the statistical analysis. **** *p* < 0.0001, *** *p* < 0.001, ** *p* < 0.01, * *p* < 0.05.

Our findings demonstrated that serum levels of all biomarkers analyzed were significantly increased in the ZIKV-infected groups compared with control groups (Figure 1). However, when the comparison was performed between ZIKV-infected groups, the Pr ZIKV+ women showed serum levels remarkably higher than the NPr ZIK+ women.

In case of IL-1β and IL-18, the serum levels were physiologically higher in Pr ZIKV− in comparison with NPr ZIKV− (*p* < 0.001), while the levels of NLRP3 were lower in Pr ZIKV− in comparison with all other groups. The results showed that acute ZIKV infection induces more intensive GDF3, NLRP3, IL-1β, and IL-18 expression in the context of pregnancy (Figure 1).

### 3.2. The ZIKV Virus Infection Induces High Expression of IL-18 during the Third Trimester of Pregnancy

**Figure 2:**
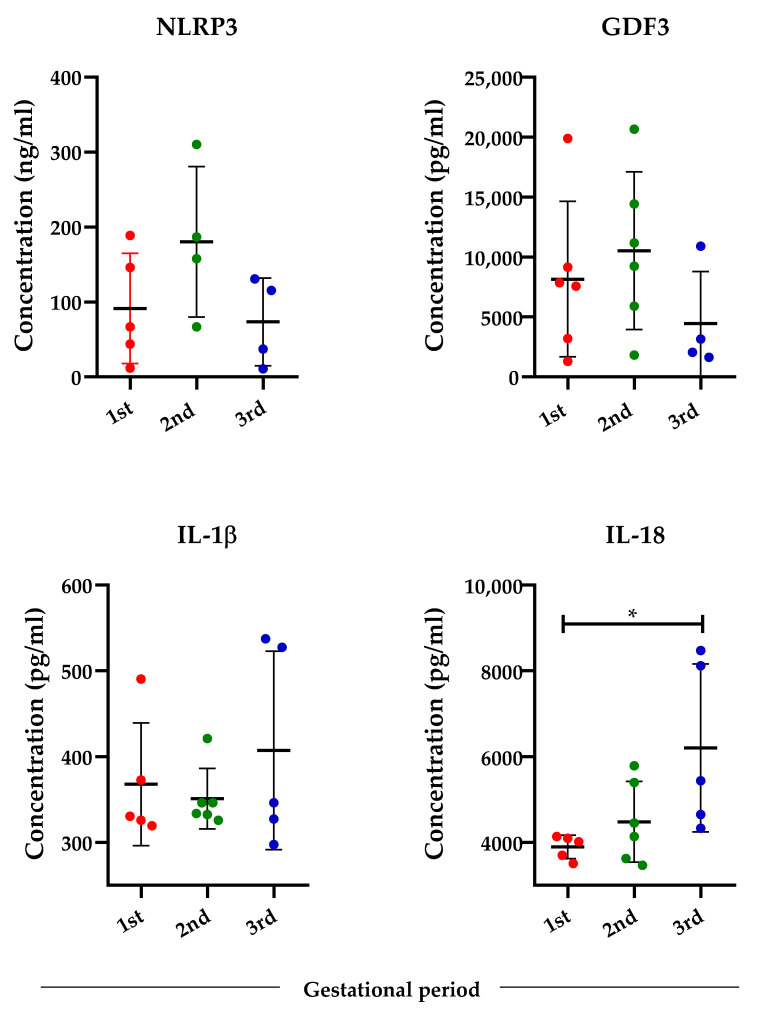
Serum analysis of GDF-3 and inflammasome-related markers according to the gestational period in the Pr ZIKV+ group. Analysis of serum concentration of NLRP3, GDF3, IL-1β, and IL-18 in the first (1st, *n* = 6), second (2nd, *n* = 6), and third (3rd; *n* = 6) trimester of pregnancy. One-Way ANOVA with Tukey’s multiple comparison test. * *p* < 0.5.

Since pregnancy undergoes distinct immunological phases characterized by specific biological processes, we next evaluated the levels of NLRP3, IL-1β, IL-18, and GDF3 in Pr ZIKV+ women at different gestational periods (Figure 2). Our findings showed no statistically significant difference between the serum levels according to gestational trimester, except IL-18 which was higher in the third trimester in comparison with the first trimester of pregnancy (*p* = 0.029).

### 3.3. GDF3 and NLRP3 Markers Are Positively Correlated in ZIKV-Infected Pregnant Women

**Figure 3:**
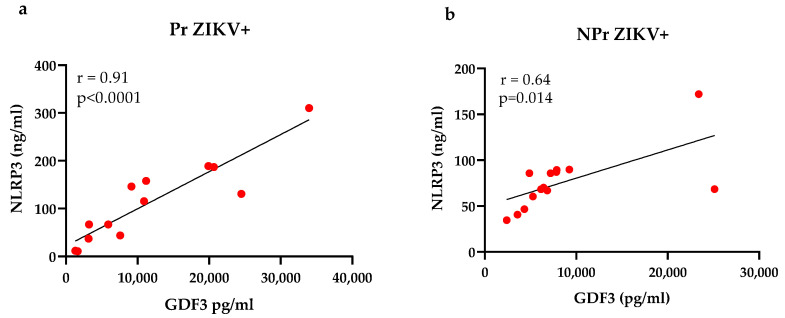
Correlation analysis between NLRP3 and GDF3 in ZIKV-infected groups. (**a**) Strong positive correlation (r = 0.91; *p* < 0.0001) in the Pr ZIKV+ group; (**b**) moderate positive correlation (r = 0.64; *p* = 0.014) in the NPr ZIKV+ group (**b**). The Pearson’s test was applied to perform correlation analysis. r > +0.7 refers to strong positive correlation.

Given that NLRP3, GDF3, IL-1β, and IL-18 were increased in the ZIKV-infected groups, next we evaluated the correlation between those markers. Using bivariate analysis, we found that GDF3 and NLRP3 are strong positively correlated in the Pr ZIKV+ group (r = 0.91, *p* < 0.0001) (Figure 3a), but moderately correlated in the NPr ZIKV+ group (r = 0.68 *p* = 0.014) (Figure 3b).

**Figure 4:**
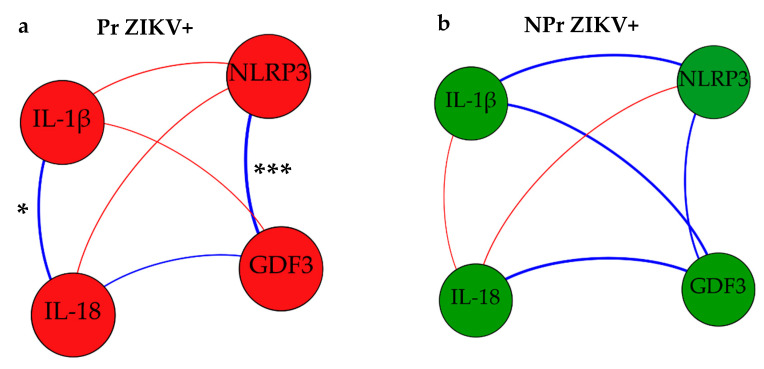
Multivariate correlation analysis of NLRP3, GDF3, IL-1β, and IL-18 in ZIKV-infected groups. (**a**) correlation among ZIKV-infected pregnant women (Pr ZIKV+); (**b**) correlation among ZIKV-infected non-pregnant women (NPr ZIKV+). Thick lines indicate strong correlation and thin lines indicate weak correlation. Blue lines refer to a positive correlation and red lines refer to a negative correlation. *** *p* < 0.001, * *p* < 0.05.

When we performed multivariate correlation analysis, both GDF3 and NLRP3 were still positively correlated (*p* < 0.001), just as IL-1β and IL-18 (*p* < 0.05) were among the Pr ZIKV+ group (Figure 4a). However, we did not observe a significant correlation between those markers in the NPr ZIKV+ group (Figure 4b).

## 4. Discussion

The immunopathogenesis of ZIKV-related adverse pregnancy outcomes remains poorly understood. In this study, we demonstrated that acute ZIKV virus infection during pregnancy elicits overexpression of NLRP3, IL-1β, IL-18, and GDF3 serum makers.

Altered inflammatory responses during pregnancy can result in severe clinical manifestations. As reported earlier, higher expression of NLRP3 is linked to negative pregnancy outcomes [20,21]. In addition, NLRP3 plays a pivotal role in the development of HELLP syndrome (hemolysis, elevated liver enzymes, and low platelet count), a serious complication that may result in maternal death [22]. The pathophysiology of this syndrome is associated with the extrinsic pathway of the coagulation cascade and hepatocyte pyroptosis mediated by NLRP3, IL-1β, and IL-18. [23,24]. Our findings showed that the serum levels of NLRP3, IL-1β, and IL-18 were increased among ZIKV-infected study groups, but significantly higher in pregnant women. Overexpression of NLRP3, IL-1β, and IL-18 immune mediators may put fetuses at high risk by increasing the possibility of central nervous system damage in neonates [25]. Besides brain damage, elevated serum levels of IL-1β are also involved in the induction of preterm labor and abnormal antenatal lung growth [26,27]. However, we did not longitudinally follow the study population to evaluate the impact of the serum raising of these markers on the fetal, neonatal, and infant outcomes.

The highest levels of GDF3 were also found among ZIKV-infected pregnant women. Although the association between GDF3 and ZIKV pathogenesis is unknown, high serum levels of GDF3 have been linked to skeletal and congenital anomalies such as microphthalmia, iris, and retinal colobomas [28,29]. A positive correlation between NLRP3 and GDF3 has been suggested in mice, because GDF3 depletion inhibited the activation of NLRP3, which resulted in the decrease of IL-1β expression in bone marrow-derived macrophages. Likewise, the depletion of NLRP3 led to a low expression of GDF3 [30]. In this study, we also observed a positive correlation between GDF3 and NLRP3 in ZIKV-infected individuals, especially among pregnant women, which may contribute to the development of ZIKV-associated birth defects. Nevertheless, more all-embracing and longitudinal studies with a larger number of participants must be performed to better understand this correlation and its impact on pregnancy outcomes.

The maternal immune system changes during all stages of pregnancy in a highly orchestrated manner to maintain gestation in balance [31]. Getting an infection during pregnancy disrupts this immunological equilibrium, leading to several maternal and fetus health complications [32]. Thus, we assessed the serum levels of GDF3, NLRP3, IL-1β, and IL-18 in ZIKV-infected pregnant women at each gestational trimester. The serum levels of IL-18 were significantly increased in the third trimester of pregnancy compared with the first trimester.

In normal pregnancy, the T helper type 2 (Th2) response predominates in the second and the majority of the third trimester [33]. Switching from a cytokine Th2 response to a type 1 cytokine T response (Th1) during these gestation stages triggers cell-mediated toxicity that can lead to spontaneous abortion [34]. In addition, IL-18 acts synergistically with IL-12 to induce the Th1 immune response [35,36]. This immunological imbalance due to IL-18 overexpression can lead to placental apoptosis and premature rupture of the amniotic membrane [37]. Our findings showed that ZIKV infection upregulates the expression of IL-18, especially in the third gestational trimester, which can put the pregnancy at high risk.

Although our results are of primary importance in describing the profile of GDF3 and ZIKV-induced inflammatory markers during pregnancy, the study population was relatively small. However, the study groups were paired with caution to minimize the effects of potential issues with confounders. In addition, the samples were collected and analyzed at one point across the study population, which limited our conclusions. Apart from these limitations, the present study broadens existing scientific information about the dynamics of immune modulation triggered by ZIKV infection during pregnancy. The raising of maternal serum level of these mediators and their immunological role in the pathogenesis of ZIKV can pose serious health threats to the mother or her fetus, particularly with respect to fetal neural development.

## 5. Conclusions

In conclusion, this study demonstrated that acute ZIKV infection during pregnancy triggers overexpression of the growth factor GDF3 and the inflammasome-related factors NLRP3, IL-1β, and IL-18. The GDF3 and NLRP3 are positively correlated in ZIKV-infected pregnant women. Despite the small sample size, our findings raise the possibility of these factors being considered predictive biomarkers of maternal, fetal, and neonatal adverse clinical outcomes associated with gestational ZIKV infection. Thus, our results provide new insights into the pathogenesis of ZIKV infection in the context of pregnancy.

## Data Availability

Not applicable.
